# São Paulo Advanced School of Comparative Oncology (ESPCA)

**DOI:** 10.1186/1753-6561-7-S2-A1

**Published:** 2013-04-04

**Authors:** Silvia Regina Rogatto

**Affiliations:** 1 Faculty of Medicine, São Paulo State University, UNESP, Botucatu, SP, Brazil; 2 International Center of Research and Training (CIPE), Hospital A.C. Camargo, São Paulo, SP, Brazil

## 

Comparative Oncology, the theme of this School, is a new area of research and has undergone a special development over the past decades. The focus in this area has increasingly attracted the attention of researchers who are interested in etiopathogenesis, tumor biology, morphogenesis, epidemiology, genomics and cancer therapeutics. Comparative Oncology integrates the study of naturally occurring cancers in animals into studies of human cancer. These studies have enormous potential to discover novel treatments for human beings by treating pet animals, specially dogs and cats with naturally occurring cancer and that share similarities with human cancer genes.

The goal of the São Paulo Advanced School of Comparative Oncology was to promote the consolidation and expansion of basic and applied cancer research in the State of São Paulo. The international exchange of technological and scientific innovation aims to establish a globally competitive hub for talented researchers in this new area.

The ESPCA was attended by 32 speakers from different countries including Brazil, Canada, France, USA, Japan, Netherlands, Germany, Finland, and UK. The School provided an invaluable opportunity for networking and fruitful contacts with cancer researchers working with human and animals.

During the event it was created a Facebook page, on which photos and news concerning the lectures and interviews were daily updated. A journalist was hired to interview the lecturers and participants. Videos were also uploaded to an Youtube account, and the link was shared on the Facebook page. The official website of the School is http://www.comparativeoncologyespca.org/.

